# Chancre

**Published:** 1886-07

**Authors:** 


Chancre.—Von Hebra, of Vienna, successfully treats chancre in four or five days by the topical use of salicylic acid. The method consists in thoroughly cleansing the parts with tepid water and soap, especially where lead, zinc or silver has been employed, covering the sore with salicylic acid powder, and retaining it in place with adhesive straps. The dressings are renewed once or twice a day, as occasion requires. The advantage claimed for this method is the prevention of chancrous bubo.—*Medical Brief.*

---

The Medical Department of the University of New York has recently received a gift of $100,000, to be devoted to the construction of a laboratory building, to be known as the Loomis Laboratory.

